# Iron dyshomeostasis, lipid peroxidation and perturbed expression of cystine/glutamate antiporter in Alzheimer’s disease: Evidence of ferroptosis

**DOI:** 10.1016/j.redox.2020.101494

**Published:** 2020-03-05

**Authors:** Azhaar Ashraf, Jérôme Jeandriens, Harold G. Parkes, Po-Wah So

**Affiliations:** a Department of Neuroimaging, Institute of Psychiatry, Psychology and Neuroscience, King’s College London, London, United Kingdom; b Department of Human Biology and Toxicology, Faculty of Medicine, University of Mons, Place du Parc 20, Mons, Belgium

**Keywords:** Ferroptosis, Iron dyshomeostasis, Lipid peroxidation, Glutamate/cystine antiporter, Excitotoxicity, Alzheimer’s disease

## Abstract

Iron dyshomeostasis is implicated in Alzheimer’s disease (AD) alongside β-amyloid and tau pathologies. Despite the recent discovery of ferroptosis, an iron-dependent form cell death, hitherto, *in vivo* evidence of ferroptosis in AD is lacking. The present study uniquely adopts an integrated multi-disciplinary approach, combining protein (Western blot) and elemental analysis (total reflection X-ray fluorescence) with metabolomics (^1^H nuclear magnetic resonance spectroscopy) to identify iron dyshomeostasis and ferroptosis, and possible novel interactions with metabolic dysfunction in age-matched male cognitively normal (CN) and AD post-mortem brain tissue (n = 7/group). Statistical analysis was used to compute differences between CN and AD, and to examine associations between proteins, elements and/or metabolites. Iron dyshomeostasis with elevated levels of ferritin, in the absence of increased elemental iron, was observed in AD. Moreover, AD was characterised by enhanced expression of the light-chain subunit of the cystine/glutamate transporter (xCT) and lipid peroxidation, reminiscent of ferroptosis, alongside an augmented excitatory glutamate to inhibitory GABA ratio. Protein, element and metabolite associations also greatly differed between CN and AD suggesting widespread metabolic dysregulation in AD. We demonstrate iron dyshomeostasis, upregulated xCT (impaired glutathione metabolism) and lipid peroxidation in AD, suggesting anti-ferroptotic therapies may be efficacious in AD.

## 1 Introduction

Prevailing evidence suggests iron (metal) dyshomeostasis as a contributing factor in Alzheimer’s disease (AD) pathogenesis, alongside amyloid plaques and tau tangles [[1], [2], [3]]. Clinical trials aimed at attenuating brain amyloid levels have failed to halt AD progression [4] suggesting other factors at play. Indeed, despite being amyloid-Positron Emission Tomography (PET)-positive, suggestive of a diagnosis of AD, 30% of individuals remain cognitively normal (CN) [5]. Iron dyshomeostasis has been proposed to be an additional factor in AD pathology as severe cognitive decline was shown to correlate with an elevated brain iron signal in amyloid-PET positive individuals [6].

Brain iron levels undergo strict regulation through import (transferrin or non-transferrin mediated) and export mechanisms (involving ceruloplasmin/ferroportin, Cp/Fpn), while intracellular iron is sequestered by ferritin [[7], [8], [9]]. The perturbed brain iron regulation in AD enables redox-active ferrous iron to both generate hydroxyl free radicals in the Fenton reaction and induce/enhance neuroinflammation, contributing to oxidative stress and neurodegeneration possibly via an iron-dependent cell death called ferroptosis [6,[10], [11], [12], [13]]. During the process of ferroptosis, iron-induced lipid peroxidation causes catastrophic membrane rupture in conditions of diminished activity of a lipid repair enzyme, glutathione peroxidase 4 (GPX4) [14]. GPX4 utilises glutathione as a cofactor, with cysteine availability being the rate limiting factor in glutathione synthesis. The cystine/glutamate transporter (X_c_^-^) uptakes cystine (oxidised cysteine) into the cell in exchange for glutamate. Inhibition of X_c_^-^ depletes glutathione levels and impairs GPX4 activity, thereby increases lipid peroxidation. Certain membrane lipids have been found to be oxidised during ferroptosis. Polyunsaturated fatty acids (PUFA) specifically containing arachidonic acid are esterified with CoA by Acyl-CoA synthetase long chain family member 4 (ACSL4) forming phosphatidylethanolamines [15], which are vulnerable to peroxidation by iron-dependent lipoxygenases [16].

Iron dyshomeostasis alongside impaired activity of X_c_^-^ [[17], [18], [19], [20]] and lipid peroxidation [11] are features of ferroptosis [10], which could provide successful therapeutic targets to attenuate AD. However, *in vivo* evidence of such ferroptosis-related processes in human AD is lacking and our study aims to determine the expression levels of proteins implicated in iron metabolism and ferroptosis in CN and AD brains. Total X-ray Reflection Fluorescence (TXRF) was also used to measure brain contents of iron and other elements, which we have shown to discriminate between plasma from CN and AD subjects [2]. Further, in a unique integrated, multi-disciplinary approach, protein and elemental analyses were combined with ^1^H NMR based metabolomics to identify iron dyshomeostasis and possible novel interactions with metabolic dysfunction in AD. Metabolic derangements have been observed previously by ^1^H NMR metabolomics in AD post-mortem brain tissues [21] and animal models [22]. We hypothesised that iron dyshomeostasis, augmented lipid peroxidation and impaired X_c_^-^, characteristics of ferroptosis, would be observed in AD. We also conjectured interactions within and between protein, elements/metals and metabolite networks to differ between CN and AD.

## 2 Material and methods

### 2.1 Samples

**Fig. 1:**
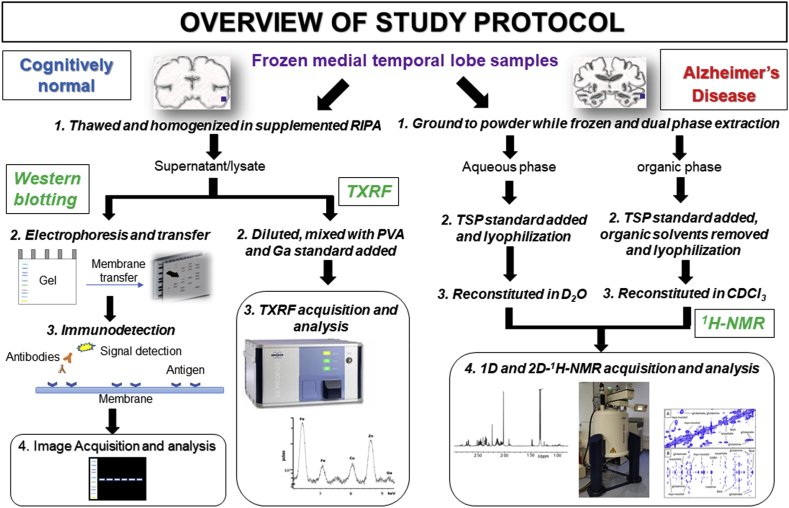
Overview of multidisciplinary techniques used in study protocol involving western blotting, total X-ray reflection fluorescence (TXRF) and ^1^H nuclear magnetic resonance (NMR) spectroscopy of human medial temporal cortical post-mortem tissue. Abbreviations: trimethylsilyl-[2,2,3,3,-^2^H_4_]-propionate (TSP), deuterium oxide (D_2_O), deuterochloroform (CDCl_3_) and radioimmunoprecipitation assay buffer (RIPA).

Frozen samples of human medial temporal gyri were obtained from the London Neurodegenerative Diseases Brain Bank (LNDBB, Brains for Dementia research) with ethical approval (London – City and East NRES committee 08/H0704/128 + 5). Samples were obtained from male CN subjects with no history of dementia and male AD patients (n = 7/group) with clinically and pathological confirmed AD (Braak Stage 6; Table 1). An overview of the study protocol is shown in Fig. 1.

**Table 1:** Demographic details of cognitively normal (CN) and Alzheimer’s disease (AD) subjects in this study. The post-mortem delay (PMD) and post-mortem interval (PMI) are the time intervals between death and brain removal, and between death and when the brain was frozen, respectively. Age, post-mortem delay (PMD) and post-mortem interval (PMI) were similar between the two groups.

I.D.	Diagnosis	Age (years)	PMD (h)	PMI (h)	Neuropathology notes
1	Cognitively normal	96	43	68	Braak Stage II - Pathological changes consistent with aging
2		82	26	27	Modified Braak Stage II – Very mild AD pathology
3		77	11	11	Normal
4		73	23	23	Normal
5		74	22.5	24	Modified Braak Stage I with mild amyloid angiopathy
6		91	47	51	Braak Stage II – pathological changes consistent with normal aging
7		80	31	54	Braak Stage II – pathological changes consistent with normal aging
mean ± standard deviation		82 ± 9	29 ± 12	37 ± 19	
8	Alzheimer’s disease	72	61	89	Modified Braak Stage VI with mild amyloid angiopathy
9		81	30	58	Modified Braak Stage VI with mild amyloid angiopathy
10		62	23	23	Modified Braak Stage VI with mild amyloid angiopathy
11		79	20	24	Modified Braak Stage VI with moderate amyloid angiopathy
12		70	20	20	Modified Braak Stage VI with extensive amyloid angiopathy
13		76	90	114	Braak Stage VI with TDP43 pathology limited to amygdala
14		90	44	69	Braak Stage VI - CERAD age related plaque score C (definite)
mean ± standard deviation		76 ± 9	41 ± 26	57 ± 34

### 2.2 Western blotting

#### 2.2.1 Sample preparation

Brain tissues (~33 mg) were extracted with 500 μl radioimmunoprecipitation assay buffer (RIPA; 1% Triton X-100, 1% sodium deoxycholate, 0.1% sodium dodecyl sulphate, 150 mM NaCl and 50 mM Tris-HCl, pH 7.2; 89901, Thermofisher, UK), supplemented with protease/phosphatase/EDTA inhibitor cocktail (87785, Thermofisher, UK) and sonicated (30 s). Following centrifugation (14,800 g, 20 min, 4°C), supernatants/lysates were stored at −80°C until further analysis.

#### 2.2.2 Western blot (WB) analysis

Lysates (containing 40 μg protein, determined using a Thermo Nanodrop Spectrophotometer ND 1000) were mixed with laemmli buffer (S3401; Sigma, Poole, UK) and heated (95 °C, 5 min). Proteins were separated on Novex^TM^ Tris-glycine 4–20% gradient gels (XP04205BOX, Thermofisher) at 150 V, 90 min and then transferred onto a 0.45 μm nitrocellulose membrane (GE10600002, GE healthcare, Amersham, UK) in Tris-glycine buffer supplemented with 20% methanol (80 V, 1 h). Membranes were incubated in a blocking solution (5% milk in PBS-0.2% Tween, 1 h, ambient temperature), prior to with primary antibodies (Supplemental Table S1; overnight, 4°C) including iron-associated proteins: ferritin-light chain (FTL), ferritin-heavy chain (FTH), transferrin-receptor (TfR), divalent metal transporter 1 (DMT1), Iron Responsive Element Binding Protein 2 (IREB2), Cp, Fpn, heme-oxygenase-1 (HO-1), melanotransferrin (MTf), lactoferrin (LTf); and for ferroptosis, Nuclear factor erythroid 2-related factor 2 (Nrf2), ACSL4, light-chain subunit of X_c_^-^ (xCT), GPX4 and the lipid-peroxidation marker 4-hydroxynonenal (4-HNE). After incubation, membranes were washed (3 x 5 min, PBS-0.2% Tween) and then incubated with horse radish peroxidase (HRP)-conjugated secondary antibodies (Thermofisher; Supplemental Table S1) in 5% milk/PBS-0.2% Tween (1 h, ambient temperature). After washing (PBS-0.2% Tween, 3 x 5 min), membranes were visualised (Pierce ECL western blotting substrate, 32106; Thermofisher) and imaged (BioRad ChemiDoc MP system). Membranes were stripped using restore Western blot stripping buffer (20159; Thermofisher) and re-probed with HRP-conjugated β-actin. Band signal intensities were quantified by densitometry (ImageJ software 2.0, NIH), and normalised to β-actin.

### 2.3 Total reflection X-ray fluorescence (TXRF)

#### 2.3.1 Sample preparation

Lysates were thawed and diluted 1:4 v/v with distilled water. Polyvinyl alcohol (2 μl, 0.3 g/l water; 843871, Merck, Gillingham, UK) was added to the diluted lysate (10 μl). Gallium solution (10 μl, 440g/l; TraceCERT® gallium standard for inductive coupled plasma; 16639, Merck) was also added (200 μg/l final concentration). After thorough mixing, 10 μl of the resultant solution was centrally placed onto an acrylic sample carrier (Bruker Nano GmbH, Germany) and air dried: duplicates were prepared, readings recorded and averaged. Supplemented RIPA buffer for tissue homogenisation was also prepared for TXRF as detailed above.

#### 2.3.2 TXRF data acquisition and analysis

Samples were analysed on a TXRF spectrometer with a molybdenum tube excitation source operating at 50 kV/600 μA (S2 PICOFOX™, Bruker Nano GmbH) and data were collected over 1000 s. After inspection and elemental identification of TXRF spectra, spectra were deconvolved using PICOFOX^TM^ software. Elemental concentrations of iron, copper, zinc, calcium and phosphorus were calculated by reference to the internal gallium standard, elemental contributions from the supplemented RIPA buffer subtracted, and normalised to protein concentrations (mg/g protein).

### 2.4 ^1^H NMR spectroscopy

#### 2.4.1 Sample preparation

Frozen brain tissue (~40 mg) underwent dual phase extraction with chloroform-methanol-water to provide aqueous and organic extracts. Tissue was ground to a fine powder with a pestle in a cardice-chilled mortar and then transferred into a solvent resistant tube (Nalgene™ OakRidge centrifuge tubes, ThermoFisher) containing methanol (2 ml; AnalaR grade; VWR Chemicals, Lutterworth, UK). Deionised water (2 ml) and chloroform (2 ml; AnalaR grade; Sigma-Aldrich, Poole, UK) was then added to the tube and the sample thoroughly mixed and centrifuged (10000 g, 10 min). After centrifugation, aqueous and organic phases were collected into separate tubes. The tissue pellet was then washed/mixed with more methanol, distilled water and chloroform (2 ml of each), centrifuged and the phases again separated and pooled with the appropriate previous collections.

To each aqueous and organic phase samples, trimethylsilyl-[2,2,3,3,-^2^H_4_]-propionate (TSP; Sigma-Aldrich, Poole, UK), a concentration and chemical shift (δ) reference standard was added (10 μl, 2.0 mg/ml deuterium oxide, Sigma-Aldrich), the solution mixed and then lyophilized. Prior to lyophilisation, methanol and chloroform were removed from the organic phase by a stream of nitrogen gas. For ^1^H NMR, the aqueous and organic phases were reconstituted in phosphate buffer (pH 7.0, 100 μl) and deuterium oxide (500 μl), and 500 μl deuterochloroform, respectively, transferred to a 5 mm NMR tube (Wilmad 507-PP, Apollo Scientific, UK) and stored at −20 °C prior to analysis.

#### 2.4.2 ^1^H NMR acquisition and analysis

1D and 2D ^1^H NMR were performed on a Bruker Avance III NMR spectrometer (Bruker Biospin, Karlsruhe, Germany) operating at 500 MHz ^1^H frequency.

##### 2.4.2.1 1D-^1^H NMR

**Fig. 2:**
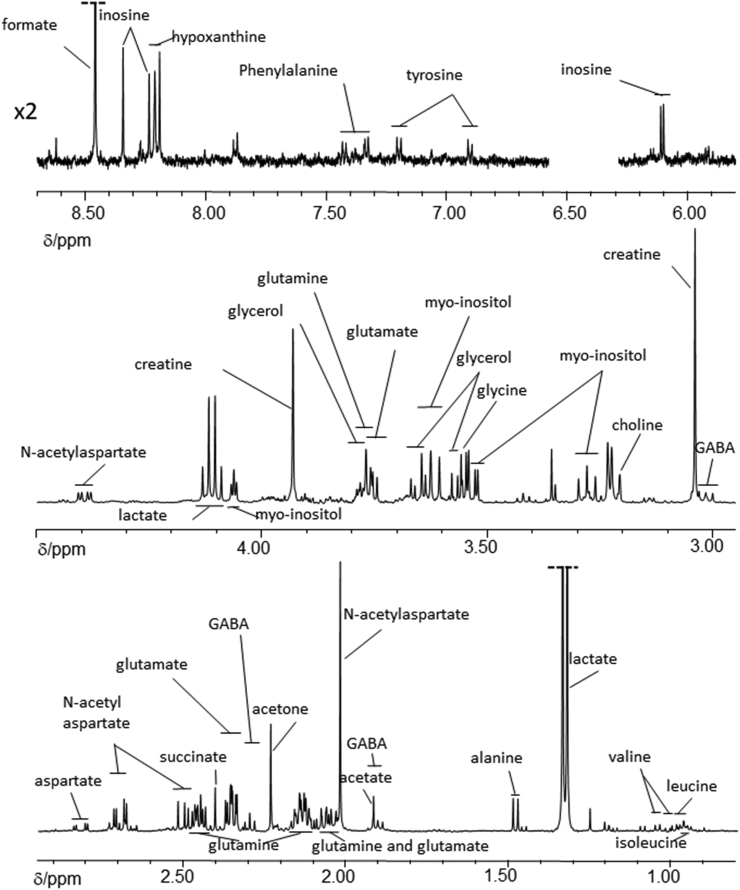
Typical partial ^1^H nuclear magnetic resonance spectrum of the δ 0.80 – 8.70 ppm region (excluding δ 4.50 - 5.80 ppm) of the aqueous phase obtained following dual phase extraction with chloroform, methanol and water of medial temporal cortex of a cognitively normal subject. Resonances from formate and lactate have been truncated. Assignments are based on Chenomx™, literature values (see Supplemental) and from ^1^H-JRES and ^1^H-^1^H COSY data (see Fig. 4, Fig. 5). Abbreviation: γ-aminobutyrate (GABA).

Aqueous phase: Spectra for aqueous extracts of brain were acquired using a 1D-^1^H-NOESY sequence with presaturation to attenuate the water resonance. Fully T1-relaxed spectra were collected with a relaxation delay of 2.00 s and acquisition time of 5.46 s. In total, 256 scans were collected with four dummy scans into 64k data points and 6000 Hz spectral width. Spectra were processed using Topspin (v 3.5, Bruker Biospin, Karlsruhe, Germany) with application of 0.2 Hz exponential line broadening, baseline corrected and referenced to trimethylsilyl-[2,2,3,3,-^2^H_4_]-propionate (TSP; Sigma-Aldrich, Poole, UK): a typical ^1^H NMR spectrum of the aqueous fraction is shown in Fig. 2. Using Chenomx™ (v8.4; Edmonton, Canada), the following metabolites were identified and quantified (mmol/g wet tissue weight): acetate, alanine, aspartate, carnitine, choline, creatine, formate, fumarate, GABA (γ-aminobutyrate), glutamate, glutamine, glycine, glycerol, hypoxanthine, inosine, isoleucine, leucine, myo-inositol, N-acetylaspartate (NAA), phenylalanine, succinate, tyrosine and valine. ^1^H-J-resolved (JRES) and ^1^H-^1^H correlation NMR spectroscopy (COSY) were also performed to aid/confirm metabolite identification (see below).

**Fig. 3:**
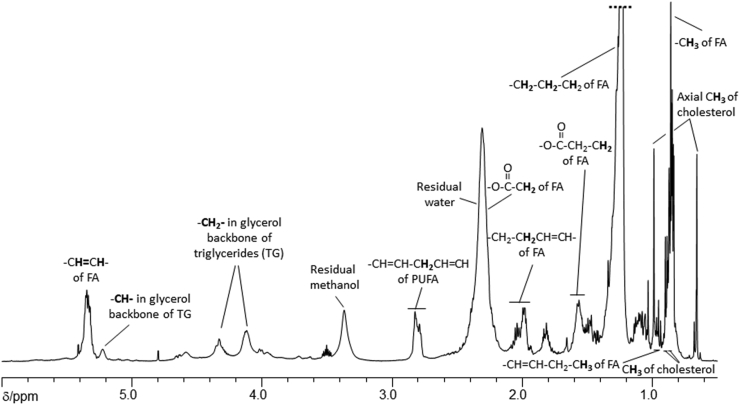
Typical partial ^1^H nuclear magnetic resonance spectrum of the δ 0.75 – 6.00 ppm region of the organic phase obtained following dual phase extraction with chloroform, methanol and water of tissue from the medial temporal lobe of a cognitively normal subject. The overlapping methylene resonances from the fatty acid chains have been truncated. Assignments are based on literature values. Abbreviations: fatty acid chains (FA), polyunsaturated fatty acids (PUFA) and triglycerides (TG).

Organic phase: 1D-^1^H NMR spectra were collected using a pulse-collect sequence and again fully T1-relaxed spectra was collected but with a relaxation delay of 2.00 s and acquisition time of 5.03 s. In total, 128 scans were collected with four dummy scans into 64k data points and 6510 Hz spectral width. Spectra were processed as for the aqueous samples using Topspin (v 3.5, Bruker Biospin) and again referenced to TSP, a typical ^1^H NMR spectrum of the organic phase is shown in Fig. 3. Characteristic resonances of fatty acids, PUFAs, triglycerides and cholesterol were identified and quantified (μmol/g wet tissue weight).

##### 2.4.2.2 2D-NMR

^1^H-^1^H Correlation spectroscopy (COSY) and ^1^H-J-resolved (JRES) NMR were also performed to aid/confirm metabolite identification.

**Fig. 4:**
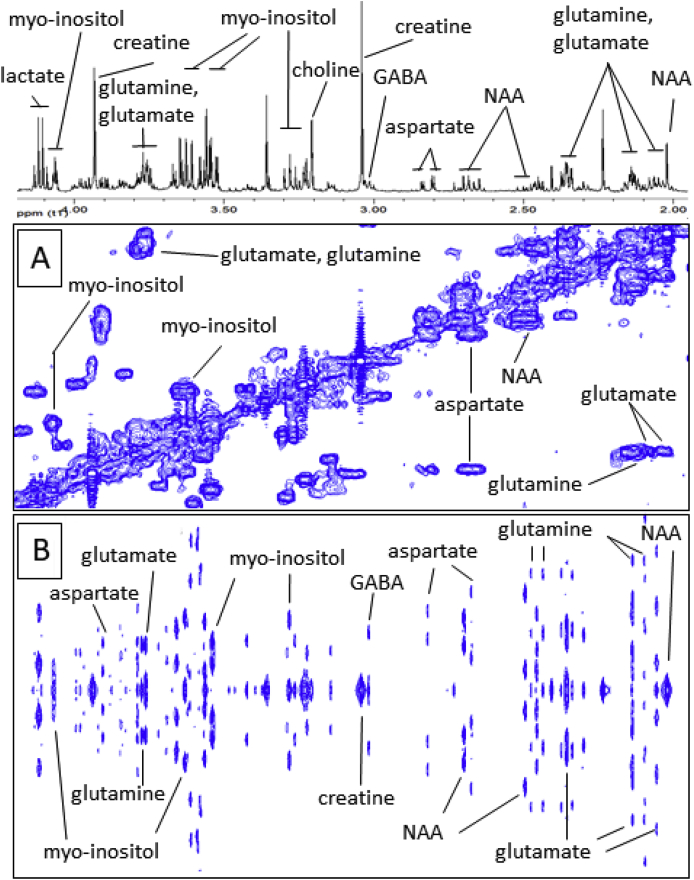
Typical partial (A) ^1^H-^1^H COSY and (B) ^1^H-JRES nuclear magnetic resonance (NMR) spectra (with the corresponding 1D-^1^H NMR spectrum) of an aqueous phase sample obtained from dual phase extraction of human medial temporal gyrus tissue. Abbreviations include: N-acetyl-aspartate (NAA) and γ-aminobutyric acid (GABA).

**Fig. 5:**
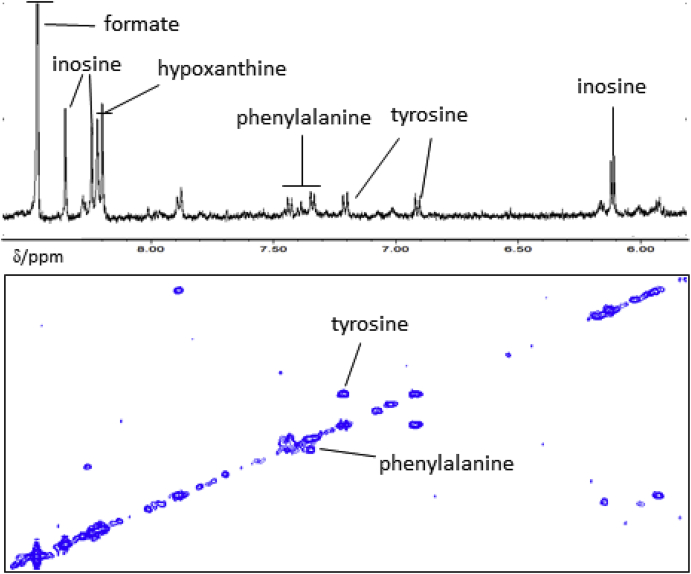
Typical partial ^1^H-^1^H-COSY nuclear magnetic resonance (NMR) spectrum (with the corresponding 1D-^1^H NMR spectrum) of an aqueous phase sample obtained from dual phase extraction of human medial temporal gyrus tissue.

^1^H-^1^H COSY NMR: COSY-NMR is used to determine correlations through chemical bonds and aids to determine resonances that are mutually coupled, usually up to four bonds. COSY data is plotted as a 2D, both axes being frequency axes, and “cross peaks” appear away from the diagonal axis when protons are coupled to each other. For the aqueous phase collected following dual phase extraction of medial temporal cortical tissue, a phase-sensitive COSY sequence was used with presaturation of the water resonance during the 2 s relaxation delay. Gradient pulses were used for selection of cross peaks, 256 increments of 4k data points were collected over a 6000 Hz sweep width, with 64 scans per increment. Spectra were zero-filled to 4k datapoints and a sine window function applied in both dimensions prior to Fourier transformation. Typical COSY NMR spectra of the aqueous phase are shown in Fig. 4, Fig. 5. For the organic phase, a phase-sensitive but double quantum filter, COSY pulse sequence was used. Again gradient pulses were used for cross peak selection, 256 increments of 2k data points collected over a 6510 Hz sweep width, with 40 scans per increment collected and a relaxation delay of 1.5 s. Spectra were zero filled to 4k points and a sine function was applied in both dimensions prior to Fourier transformation.

^1^H J-resolved NMR spectroscopy (JRES): 2D JRES is another 2D-NMR technique that aids identification of resonance multiplicities. JRES NMR spectra has the chemical shift along one axis and the proton-proton coupling along the other axis. Spectra were carried out using a JRES sequence with presaturation of the water resonance during the 2 s relaxation delay. Gradient pulses were used for selection, a total of 80 increments of 16 k points over a sweep width 6000 Hz and 32 scans per increment were collected. Spectra were zero filled to 16 k and a sine function was applied in both dimensions prior to Fourier transformation. Typical JRES NMR spectra of different regions of the spectrum of an aqueous phase sample obtained following dual phase extraction of medial temporal cortical tissues are shown in Fig. 4, Fig. 5.

### 2.5 Statistical analysis

Statistical analysis was performed using IBSS SPSS statistics 24. A two-tailed *t*-test was used to compute differences between CN and AD and Pearson’s correlation analysis to examine association between variables. Data normality was assessed using Q-Q, residual and homoscedasticity plots: values violating these assumptions were identified as outliers and excluded from analysis. Values are recorded as mean ± standard deviation (S.D.) and t-values and degrees of freedom (df) provided. Significance was set at p≤0.05, with *, **, *** and ^ns^, being p<0.05, p<0.01, p<0.005 and not significant, respectively.

## 3 Results

The authors confirm that the data supporting the findings of this study are available within the article and the supplemental.

### 3.1 Western blot

**Fig. 6:**
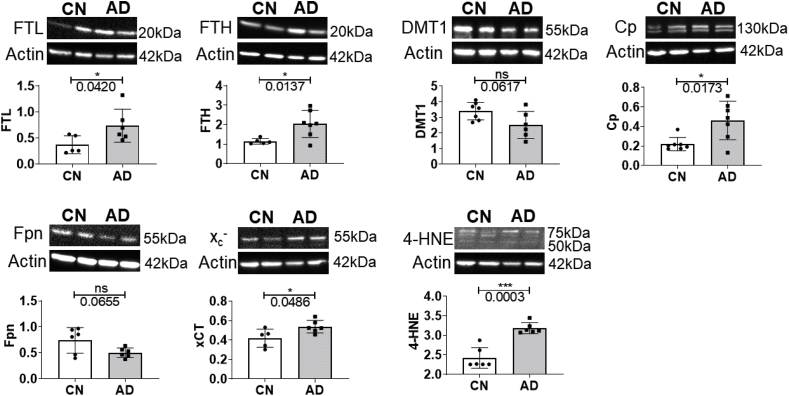
Expression levels of ferritin-light chain (FTL), ferritin-heavy chain (FTH), ceruloplasmin (Cp), ferroportin (Fpn), cystine/glutamate transporter (Xc-) and 4-hydroxynonenal (4-HNE) adducts in the medial temporal cortex of cognitively normal (CN) and Alzheimer’s disease (AD) subjects, along with representative western blots. Graph presents individual values and the mean ± standard deviation. A p-value ≤ 0.05 was considered significant; with * and ***, p < 0.05 and 0.005, respectively.

Western blot analysis suggested iron dyshomeostasis, with significantly increased expression of iron-storage proteins, FTL (p=0.0420) and FTH (p=0.0137), in AD compared to CN (Supplemental Table S2, Fig. 6). Ferroxidase Cp expression was augmented in AD brains (p=0.0173), whilst decreasing trends were observed for DMT1 (p=0.0617) and iron-exporter Fpn expression (p=0.0655). Moreover, xCT expression (p=0.0486) and the lipid peroxidation product, 4-HNE (p=0.0003) were significantly elevated in AD, implicating ferroptosis as a mechanism operant in AD. The proteins: IREB2, TfR, HO-1, MTf, LTf, Nrf2, ACSL4 and GPX4 were not significantly altered in AD compared to CN (Supplemental Table S2, Supplemental Fig. S1).

### 3.2 TXRF

**Fig. 7:**
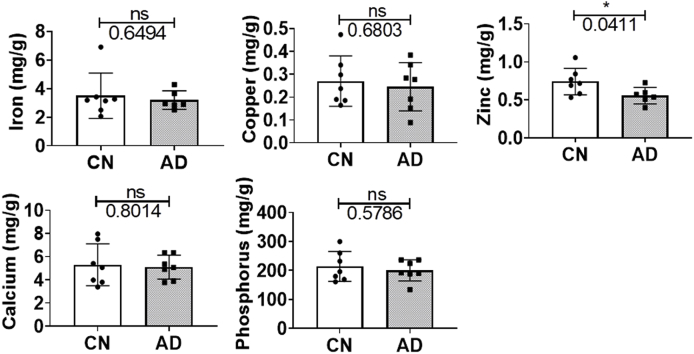
Total Reflection X-ray Fluorescence (TXRF) measurements of elemental concentrations of iron, copper, zinc, calcium and phosphorus in the medial temporal cortex of cognitively normal (CN) and Alzheimer’s disease (AD) subjects. Graph shows individual values and the mean ± standard deviation mg/g protein. The significance threshold was set at *p ≤ 0.05. Abbreviation: not significant, ns.

Iron dyshomeostasis may alter brain iron levels and so TXRF was used to quantify elemental iron concentrations. Elemental copper, zinc, calcium and phosphorus levels were also measured as we have previously found alterations in their plasma levels in AD [2]. The iron levels in the temporal cortex were similar (p=0.6494; Table 2) between groups but zinc was significantly diminished in AD compared to CN (p=0.0411; Table 2; Fig. 7). Meanwhile, elemental concentrations of copper (p=0.5158), calcium (p=0.8136) and phosphorus (p=0.4356; Table 2) were comparable between CN and AD.

**Table 2:** Elemental concentrations (mg/g protein) in lysates prepared from homogenisation of medial temporal cortical tissue from cognitively normal (CN) and Alzheimer’s disease (AD) subjects. Data are presented as mean ± standard deviation (sample size). Significance was set at *p ≤ 0.05, and t-value and degrees of freedom (df) shown. Elemental concentrations differing significantly differently between CN and AD are in bold.

	CN(n) mg/g protein	AD(n) mg/g protein	p-value	t-value (df)
Iron	3.503 ± 1.579 (7)	3.195 ± 0.653 (6)	0.6496	0.472 (8.238)
Copper	0.270 ± 0.110 (7)	0.246 ± 0.105 (7)	0.6803	0.422 (11.98)
**Zinc**	0.742 ± 0.174 (7)	0.556 ± 0.108 (6)	0.0411*	2.338 (10.15)
Calcium	5.296 ± 1.808 (7)	5.092 ± 1.032 (7)	0.1977	0.259 (9.531)
Phosphorus	214.00 ± 52.00 (7)	200.00 ± 36.00 (7)	0.5786	0.573 (10.77)

### 3.3 ^1^H NMR spectroscopy

Metals are often essential cofactors in metabolic reactions and can modulate metabolism. Thus, ^1^H-NMR-based metabolomics was used to assess metabolite levels, particularly those involved in neuronal function.

**Fig. 8:**
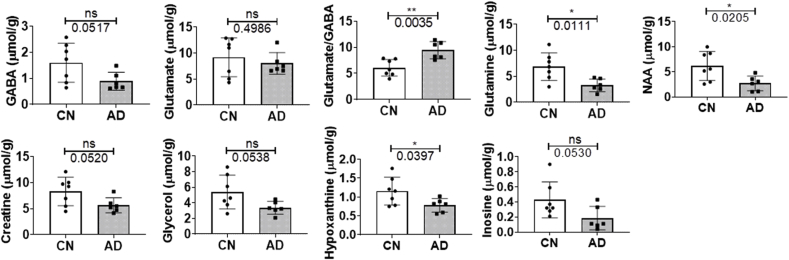
Levels of selected metabolites obtained from dual phase extraction of medial temporal cortical tissues from cognitively normal (CN) and Alzheimer’s disease (AD) subjects. Graphs shows individual values and the mean ± standard deviation μmol/g wet tissue. Significance was set at a threshold of p ≤ 0.05, with * and ** being p < 0.05 and 0.01, respectively. Abbreviation: not significant, ns.

Expectedly, GABA (p=0.0517) showed a trend of decrease in AD post-mortem tissue (Fig. 8) but glutamate levels were similar in AD and CN tissues (p=0.4986). However, the glutamate to GABA, or excitatory: inhibitory ratio, was significantly enhanced in AD, reminiscent of increased excitotoxicity (p=0.0035). Meanwhile, glutamine (p=0.0111) and NAA (p=0.0205) were found to be significantly decreased in AD tissues (Fig. 8, Supplemental Table S3). Other metabolites, creatine (p=0.0520) and glycerol (p=0.0538), demonstrated a trend of decrease in AD (Fig. 8). Further, hypoxanthine levels were decreased in AD (p=0.0397), while a trend of decrease was apparent for inosine (p=0.0530; Fig. 8, Supplemental Table S3). The levels of fatty acids (p=0.0707), PUFA (p=0.0949), triglycerides (p=0.1570), cholesterol (p=0.4524) were similar between CN and AD groups (Supplemental Table S3).

### 3.4 Correlation analysis

**Fig. 9:**
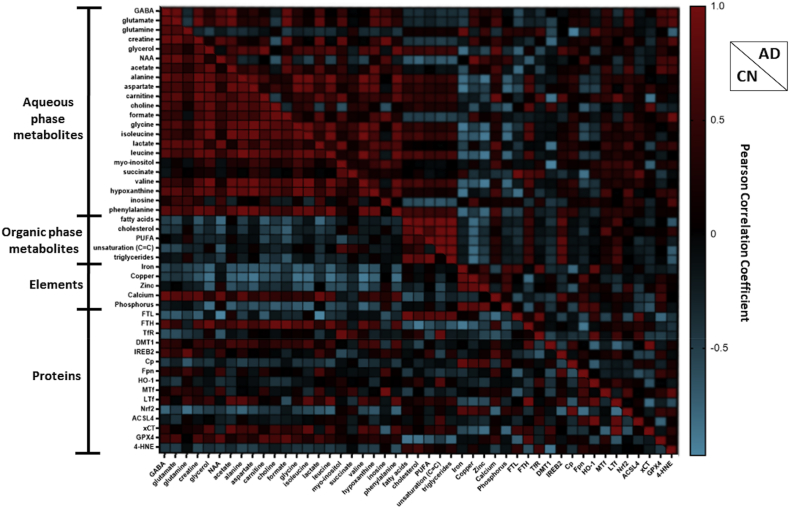
Heatmap showing the Pearson’s correlation coefficients between metabolites, metals/elements and proteins measured in the cognitively normal (CN) and Alzheimer’s disease (AD) groups. Abbreviations are: γ-aminobutyrate (GABA), N-acetylaspartate (NAA), polyunsaturated fatty acids (PUFA), ferritin-light chain (FTL), ferritin-heavy chain (FTH), transferrin-receptor (TfR), divalent metal transporter 1 (DMT1), Iron Responsive Element Binding Protein 2 (IREB2), ceruloplasmin (Cp), ferroportin (Fpn), heme-oxygenase-1 (HO-1), melanotransferrin (MTf), lactoferrin (LTf), Nuclear factor erythroid 2-related factor 2 (Nrf2), Acyl-CoA Synthetase Long Chain Family Member 4 (ACSL4), the light-subunit of the cystine/glutamate antiporter (xCT), glutathione peroxidase 4 (GPX4) and 4-hydroxynonenal (4-HNE). Note, unsaturation (C=C) refers to quantification of –CH2-CH=CH-CH2- of fatty acid chains.

Correlations within aqueous phase metabolite and element networks were more apparent in CN compared to AD but comparable for the organic phase metabolite network (Fig. 9).

**Fig. 10:**
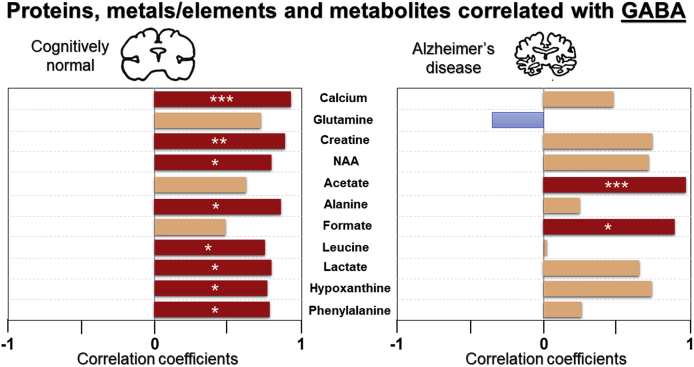
Significant and trend Pearson correlations of γ-aminobutyrate (GABA) with metals/elements and metabolites in cognitively normal and Alzheimer’s disease. Significance is set at p≤0.05, with *, ** and *** being p<0.05, <0.01 and < 0.005, respectively. Abbreviation: N-acetylaspartate (NAA).

A variation in correlation profile was observed for CN compared to AD for the aqueous and organic phase metabolites network (Fig. 9). Closer inspection shows GABA was positively associated with creatine (r=0.895, p=0.006**), glutamate (r=0.885, p=0.008**), NAA (r=0.817, p=0.025*), hypoxanthine (r=0.786, p=0.036*), phenylalanine (r=0.796, p=0.032*) and calcium (r=0.916, p=0.004***) in CN, while a trend of positive correlation was evident between GABA and glutamine (r=0.745, p=0.055^ns^; Fig. 10). Conversely, in AD subjects, a significant association was observed between GABA and glutamate (r=0.936, p=0.006**), but none were observed with creatine (r=0.777, p=0.069^ns^), NAA (r=0.750, p=0.086^ns^), hypoxanthine (r=0.757, p=0.081^ns^), phenylalanine (r=0.282, p=0.588^ns^), calcium (r=0.506, p=0.384) or glutamine (r=−0.361, p=0.482; Fig. 10).

Higher levels of fatty acids were associated with increased FTL (r=0.937, p=0.019*) but lower levels of NAA (r=−0.816, p=0.025*) and GPX4 (r=−0.880, p=0.009**) in CN but not in AD (Fig. 9). However, higher fatty acid levels were correlated to higher IREB2 (r=0.814, p=0.049*) in AD but not in CN (r=−0.073, p=0.876^ns^). Meanwhile, higher cholesterol was associated with lower levels of FTH (r=−0.902, p=0.037*) and GPX4 (r=−0.852, p=0.015) only in CN. Also, a negative correlation was observed between triglyceride and GPX4 levels (r=0.792, p=0.034*) in CN but not in AD (r=−0.033, p=0.951^ns^). Higher levels of PUFA were associated with increased IREB2 in AD (r=0.845, p=0.017*) but not in CN (r=0.484, p=0.271^ns^).

**Fig. 11:**
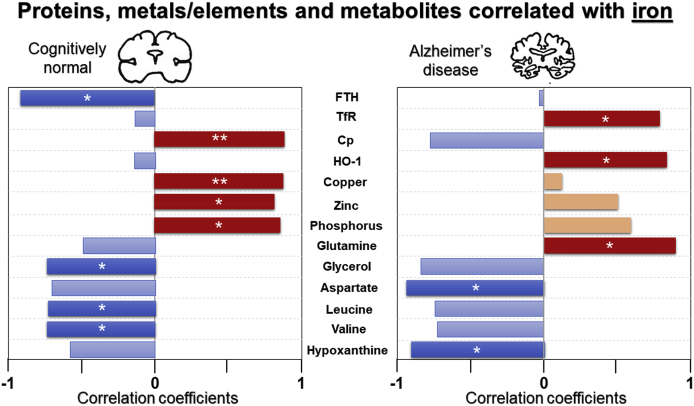
Significant and trend Pearson correlations of iron with proteins, elements/metals and metabolites in cognitively normal and Alzheimer’s disease. Significance is set at p ≤ 0.05, with * and ** being p<0.05, <0.01, respectively. Abbreviations: ferritin-heavy chain (FTH), transferrin-receptor (TfR), ceruloplasmin (Cp) and heme-oxygenase-1 (HO-1).

In CN, higher calcium was associated with higher NAA (r=0.867, p=0.011*), but lacking in AD (r=0.337, p=0.580^ns^; Fig. 9). Importantly, higher iron was associated with augmented phosphorus (r=0.862, p=0.013*), copper (r=0.879, p=0.009**), zinc (r=0.823, p=0.023*) and Cp (r=0.893, p=0.007**) in CN, but not in AD (Fig. 11). Interestingly, there was a trend of negative correlation between iron and Cp in AD (r=−0.807, p=0.052^ns^; Fig. 11). Moreover, elevated iron was significantly associated with higher glutamine (r=0.89, p=0.039*), TfR (r=0.816, p=0.048*) and HO-1 (r=0.484, p=0.033*), but lower hypoxanthine (r=−0.904, p=0.035*) in AD (Fig. 11). Similarly, higher FTL correlated to higher TfR (r=0.889, p=0.018*), and a trend of negative association between FTL and GPX4 (r=−0.804, p=0.054^ns^) in AD (Fig. 9). The ferroxidase ferritin subunit, FTH, was negatively associated with iron (r=−0.908, p=0.033*) in CN, whereas in AD, augmented FTH levels were correlated to lower Fpn levels (r=−0.865, p=0.026*; Fig. 11).

Focussing on associations with proteins involved in ferroptosis, upregulated x_c_^-^ was associated with increased DMT1 (r=0.974, p=0.005**) and lower LTf (r=−0.847, p=0.033*) in AD and not observed in CN (Fig. 9). Also, higher ACSL4 correlated to attenuated GPX4 in AD (r=−0.783, p=0.037*) but not in CN (r=−0.347, p=0.445^ns^). In the latter, elevated GPX4 was associated with increased NAA (r=0.791, p=0.034*) but absent in AD (r=0.632, p=0.178^ns^; Fig. 9). Lower 4-HNE was associated with higher DMT1 (r=−0.884, p=0.019*) in CN. However, in AD, augmented levels of 4-HNE correlated to higher levels of MTf (r=0.839, p=0.037*; Fig. 9).

## 4 Discussion

**Fig. 12:**
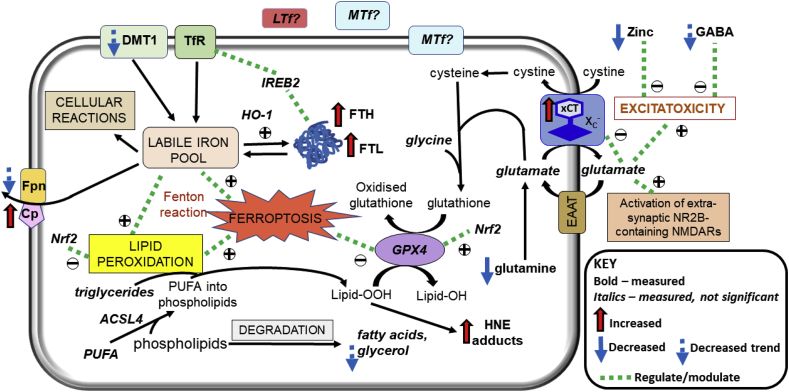
Overview of ferroptotic-associated changes in Alzheimer’s disease. Iron dyshomeostasis is observed in the form of increased ferritin levels and decreased export of iron. The consequent increases in labile iron pool enables redox-active (ferrous) iron to precipitate oxidative stress via Fenton reaction. The upregulated expression of the light-subunit (xCT) of the cystine/glutamate transporter (X_c_^-^) may contribute to excitotoxicity via NR2B-containing N-methyl-d-aspartate receptors (NMDARs), evidenced by augmented excitatory glutamate to inhibitory γ-aminobutyrate (GABA) ratio, alongside zinc-deficiency and attenuated glutamine levels, culminating in lipid peroxidation and iron-dependent cell death, ferroptosis. Abbreviations are: ferritin-light chain (FTL), ferritin-heavy chain (FTH), transferrin-receptor (TfR), divalent metal transporter 1 (DMT1), Iron Responsive Element Binding Protein 2 (IREB2), ceruloplasmin (Cp), ferroportin (Fpn), heme-oxygenase-1 (HO-1), melanotransferrin (MTf), lactoferrin (LTf), Nuclear factor erythroid 2-related factor 2 (Nrf2), Acyl-CoA Synthetase Long Chain Family Member 4 (ACSL4), glutathione peroxidase 4 (GPX4), 4-hydroxynonenal (4-HNE), polyunsaturated fatty acids (PUFA), excitatory amino acid transporter (EAAT), and lipid peroxides and alcohols (Lipid-OOH and Lipid-OH, respectively).

We demonstrate ferroptotic-like changes in the AD brain, with evidence of iron dyshomeostasis, increased expression of xCT and lipid peroxidation, co-existent with augmented excitatory glutamate: inhibitory GABA ratio. These combined metal/elemental, molecular and metabolic findings implicate oxidative stress and impaired glutathione antioxidation, concomitant with iron dyshomeostasis to be operant in AD (Fig. 12) and suggests therapies targeting ferroptosis are potentially beneficial.

AD brains exhibited augmented expression of iron-storage proteins, FTH and FTL, hinting towards an increase in the labile iron pool in AD, although elemental iron levels were comparable in CN and AD. Previously, brain iron was reported to both accumulate in AD [[6], [23]] and to be unaltered [24]. We postulate that despite elevated ferritin, ferritin’s ability to oxidise and store iron in a non-toxic but bioavailable form is rendered ineffective and may explain the dissociation between iron levels and ferritin [25,26]. Indeed, ferritin in the AD brain appears to be different from physiological ferritin, with catalytic sites available for Fenton reactions to enhance oxidative stress [27]. Augmented ferritinopathy has been observed in neuroferritinopathy [28] and ferritinopathy is required for bromoprotein BRD4 inhibitor-induced ferroptosis in cancer cells [29]. Also, ferritin aggregate formation in ferritinopathy may induce functional ferritin deficiency to accentuate iron-mediated oxidative stress [7,28,30]. Interestingly, increased FTL was associated with reduced GPX4 levels in AD in our study, suggesting dysfunctional ferritin detrimentally attenuates brain antioxidant capacity, with glutathione levels shown to be reduced in AD [31]. Although plasma glutathione has been reported to be comparable between CN and AD, lower glutathione was associated with severe cognitive impairment [32].

We observed compensatory Cp-upregulation in AD, Cp is needed to oxidise ferrous iron to ferric iron to facilitate Fpn-mediated iron export. Along with a trend of decreased Fpn expression in AD, a deficiency in cellular iron efflux is implicated as a mechanism underlying iron dyshomeostasis. In congruence with our study, Fpn is decreased in human AD and APP transgenic mouse brains and in response to ischaemia and inflammation [[33], [34], [35]], the latter inducing hepcidin-upregulation to internalise and degrade Fpn [34]. The lack of cellular iron egress contributes to an elevation in the labile ferrous iron pool, contributing to (via the Fenton reaction) lipid peroxidation [11], a signature of ferroptosis [10]. Indeed, we observed increased 4-HNE adducts, products of lipid peroxidation in AD.

Zinc exerts antioxidant effects to potentially ameliorate free-radical mediated oxidative damage [36]. Interestingly, a zinc-deficient diet resulted in iron accumulation in the liver, kidney, spleen and testes [37,38]. We observed significant decrements in brain zinc in AD, alluding to decreased antioxidant capacity and enhanced oxidative stress in a zinc-deficient brain environment [39], consistent with our previous TXRF study showing decreased plasma zinc in AD [2]. Furthermore, zinc appears to be intimately involved in the regulation of glutamatergic signalling (with ~50% of glutamatergic synapses being zinc-rich) [40,41], attenuated GABAergic signalling may arise from glutamate-induced excitotoxicity under conditions of brain zinc deficiency.

The accruing iron-dependent oxidative stress may explain our finding of upregulated-xCT in AD as a result of X_c_^-^-inhibition [42]. The X_c_^-^ antiporter comprises a light-chain subunit (xCT, SLC7A11) and a heavy-chain subunit (CD98hc, SLC3A2) [[42], [43], [44]], with the latter undertaking the simultaneous import of extracellular cystine and export of intracellular glutamate [20] (Fig. 12). Intracellular cystine is then reduced to cysteine (Fig. 12), the rate-limiting precursor for glutathione synthesis [20]. Thus, X_c_^-^-inhibition attenuates glutathione synthesis, perturbing cellular redox balance and inducing ferroptosis [42]. Oxidative stress upregulates xCT via Nrf2-upregulation [45,46], nuclear translocation of Nrf2 is required and may explain the observed upregulation of xCT, despite normal Nrf2 expression. Indirect evidence supporting xCT-upregulation in AD is indicated by increased phosphorylation of eukaryotic initiation factor 2α and activating transcriptional factor-4 expression [47]. Increased expression of xCT has also been reported in mice harbouring human APP mutations or given hippocampal injections of Aβ [17,19]. Our study demonstrates for the first time, to the best of our knowledge, increased xCT-expression in human AD. The new PET tracer for measuring X_c_^-^ function, ^18^F-5-fluoro-aminosuberic acid [48], will aid determination of whether xCT-upregulation contributes to glutamate-induced excitotoxicity since xCT is capable of transport in the absence of the heavy-chain subunit (37). We observed an augmented excitatory glutamate to inhibitory GABA ratio, suggesting enhanced glutamate-induced excitotoxicity in AD. Furthermore, excessive extracellular glutamate has been shown to inhibit X_c_^-^ and induce ferroptosis [43]. While glutamate was comparable between CN and AD in our study, ^1^H NMR is unable to differentiate between intracellular and extracellular glutamate.

MTf expression was similar in CN and AD but higher MTf was associated with increased lipid peroxidation in the latter. This is consistent with our previous report of diminished baseline cerebrospinal fluid (CSF) MTf, associated with lower hippocampal volumes and worse cognitive scores in mild-cognitive impairment (MCI) [49]. Baseline CSF MTf was lower in MCI-subjects progressing to AD compared to those remaining stable. Altered MTf metabolism and its positive association with lipid peroxidation suggests an important avenue to explore in AD pathogenesis, although the function of MTf and its exact role in iron metabolism remains elusive.

In AD, augmented ACSL4 levels were associated with lower levels of GPX4. ACSL4 is responsible for insertion of arachidonic acid (AA, a PUFA) into phospholipids, particularly phosphatidylethanolamines, contributing to formation of 4-HNE adducts/lipid peroxidation products [15,50,51]. Ablation of ACSL4 in mice reduced AA insertion into phosphatidylethanolamines and formation of 4-HNE adducts, and improved glutathione-mediated detoxification [51], consistent with our correlation analysis. ACSL4 appears to define cellular lipid composition and dictate ferroptosis-sensitivity via augmenting lipid peroxidation [15,50,51].

**Fig. 13:**
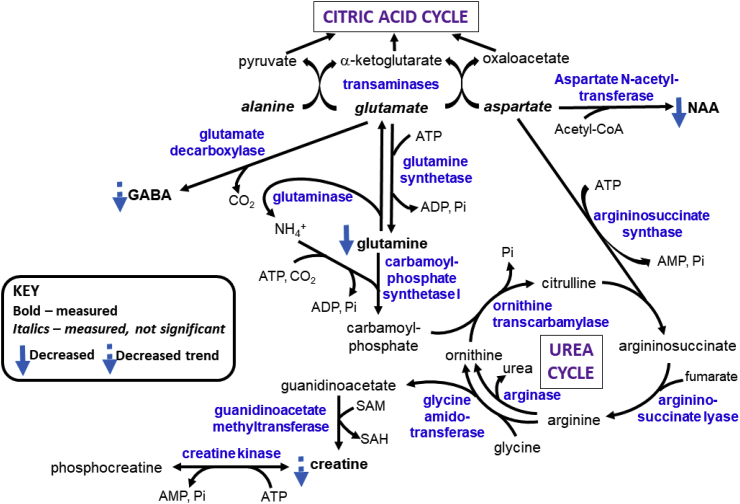
Overview of selected metabolic pathways involving glutamate, glutamine and γ-aminobutyrate (GABA). Abbreviations: N-acetylaspartate (NAA), S-adenosyl-methionine (SAM), S-adenosylhomocysteine (SAH), inorganic phosphate (Pi).

Glutamine modulates glutamatergic and GABAergic neurotransmission to protect against Aβ and hydrogen peroxide-induced oxidative stress [52]. Our depleted glutamine suggests increased glutaminolysis (conversion of glutamine to glutamate via glutaminase; Fig. 13), which may explain the increased excitatory glutamate to inhibitory GABA ratio we observed. Interestingly, glutaminolysis has been shown to regulate ferroptosis alongside transferrin [53]. In normal brains, glutamate-activation of post-synaptic receptors e.g. N-methyl-d-aspartate receptor (NMDAR), is terminated by astrocytic uptake of glutamate and then its amination to glutamine by ammonia, catalysed by glutamine synthetase (GS) (Fig. 13), protecting neurons against glutamate excitotoxicity [52,54]. The depletion of glutamine in AD brains, may be due to Aβ-mediated oxidation of GS, previously observed in MCI and AD [12]. Not only will GS-inhibition enhance excitotoxicity, accumulation of ammonia may induce astrocyte swelling and dysfunction [55]. Excess brain ammonia has been proposed as a potential neurotoxic factor in AD [56]. In the hyperammonemic state, NMDAR-mediated activation of the NO/cGMP pathway is induced with the production of nitrogen radicals and peroxynitrates [55,57], enhancing oxidative stress, which can be counteracted by interaction of glutamine with NMDAR via attenuation of NO synthesis [55,58]. The reduced brain creatine we observed in AD is consistent with compensatory increased partitioning of arginine to ornithine rather than creatine synthesis to detoxify the prevailing accruing ammonia levels (Fig. 13). However, brain creatine is mostly obtained from the liver and kidneys via the blood, although shown to be synthesised in the developing brain and requisite enzymes have been detected in glia [59]. Phosphorylation of creatine by choline kinase (CK) generates phosphocreatine, stores of which continuously and efficiently replenishes ATP via the reversible CK-catalysed reaction (Fig. 13). Notably, CK facilitates vesicular glutamate uptake and is one of the three major specifically oxidised proteins in the AD brain [60], contributing to excitotoxicity and energy deficiency. Creatine has been reported to protect rat hippocampal neurons against Aβ-toxicity by restoring energy sufficiency [61] alongside its antioxidant properties [62].

Glycerol and fatty acids can be produced from phospholipids by degradation of plasma membranes in neurodegeneration [63] (Fig. 12). Here, we report a decrease in NAA consistent with neuronal death in AD. While increased glycerol levels would be expected, we demonstrated a trend of decreased glycerol in AD, consistent with decreased glycerol, accompanied by an increase in its phosphorylated derivative, observed previously, suggesting a switch to alternative energy sources due to impaired glucose utilisation in AD [63]. Indeed, increased glutamate export resulting from xCT-upregulation may contribute to the energy deficit in AD by decreasing intracellular glutamate availability for transamination reactions to produce citric acid cycle intermediates for ATP generation (Fig. 13).

We showed that hypoxanthine levels were depressed and its precursor, inosine, to exhibit a decreasing trend. Previously, these purine metabolites were reported to be reduced in frontal cortical tissue of mild AD but unaltered/increased in the temporal cortex of severe AD [64]. Disrupted purine metabolism in AD has been demonstrated in such tissues [65] and metabolomic analyses of CSF taken at post-mortem [66] and from living subjects [67].

Since this was an exploratory study with a small sample size, p-value correction was not performed. Future subsequent studies with a larger sample size are required to confirm the findings of this preliminary study. The post-mortem approach enables direct measurement of brain iron. Whilst magnetic resonance imaging (MRI) is increasingly used to image iron *in vivo*, the strength of the MRI-iron signal is dependent on both the iron concentration and the form of iron present [25]. Our study design is original by using TXRF to directly quantify elemental (iron) concentrations in human AD brain tissue, alongside concomitant analyses of proteins and metabolites from the same subject. But the study has limitations in that we only measured iron at a single time-point and did not perform temporal staging of cortical iron levels during progression of AD. Hence, whether iron acted upstream of APP or secondary to Aβ-aggregation is unknown and the progression of cortical iron dyshomeostasis requires elucidation. Information regarding the dietary intake or excretion of iron, copper or zinc was not available. The zinc deficiency in the AD brain we observed may arise from malnutrition, commonly observed at end-stage AD [2].

We did not evaluate the oxidation state of proteins under investigation, which could serve as a useful biomarker for oxidative stress and impact elemental and metabolomic measurements. Another study limitation is the use of post-mortem human tissues for metabolomic studies. The agonal state and the inevitable delay between death and tissue processing may hamper the preservation of some metabolites whose “lifespan” may vary from seconds to a few hours. Although there was variability in the age, PMD and PMI between CN and AD, they were not statistically different. The metabolic changes we observed in AD are consistent with previous reports.

In conclusion, we demonstrate iron dyshomeostasis, upregulated xCT (perturbed glutathione metabolism) and lipid peroxidation, signatures of ferroptosis in AD, thus supporting the use of anti-ferroptotic therapies in AD. Our novel finding of xCT-upregulation suggests caution must be employed when attempting to inhibit ferroptosis by restoring glutathione antioxidant capabilities via X_c_^-^-targeting, as this may contribute to further excitotoxicity and energy deficiency in AD.

## Funding

This study was sponsored by the 10.13039/501100000268Biotechnology and Biological Sciences Research Council (BBSRC), King’s College London and Perspectum Diagnostics Ltd by funding Azhaar Ashraf’s industrial PhD studentship. Also, we would like to thank the 10.13039/100010269Wellcome Trust for funding the London Metallomics Facility (grant reference 202902/Z/16/Z) and to the 10.13039/100014461Biomedical Research Centre at 10.13039/100009362South London and Maudsley NHS Foundation Trust and King’s College London for funding costs of Chenomx™ software and TXRF consumables.

## Declaration of competing interest

The authors declare that they have no known competing financial interests or personal relationships that could have appeared to influence the work reported in this paper.
