# Supplementary material for: Iron dyshomeostasis, lipid peroxidation and perturbed expression of cystine/glutamate antiporter in Alzheimer’s disease: Evidence of ferroptosis
Source: Redox Biol. 2020 Mar 5;32:101494. doi: 10.1016/j.redox.2020.101494 (PMC7083890; doi:10.1016/j.redox.2020.101494)

**Supplemental**

**Table S1** – Details of antibodies and dilutions, and blocking media used for western blotting. Appropriate secondary antibodies used: horseradish peroxidase-conjugated anti-mouse, anti-rabbit and anti-goat secondary antibodies (62-6520, 31460, PA1-28664, respectively; Thermofisher).

| **Antibody** | **Dilution** | **Blocking medium** (PBS-Tween0.2%) |
| --- | --- | --- |
| Rabbit Ferritin light-chain (ab69090; Abcam) | 1/500 | 2% milk |
| Mouse Ferritin heavy-chain (MABC602; Merck) | 1/1000 | 2% milk |
| Mouse Transferrin receptor (13-6800; Thermofisher) | 1/1000 | 2% milk |
| Rabbit Divalent metal transporter 1 (ab123085; Abcam) | 1/1000 | 2% milk |
| Rabbit Iron Responsive Element Binding Protein 2 (PA1-16543; Thermofisher) | 1/500 | 2% BSA |
| Rabbit Ceruloplasmin (PA5-14225; Thermofisher) | 1/500 | 2% milk |
| Rabbit Ferroportin (ab78066; Abcam) | 1/1000 | 2% BSA |
| Rabbit Heme-oxygenase 1 (ab1284; Merck) | 1/1000 | 2% milk |
| Mouse Melanotransferrin (ab201922; Abcam) | 1/500 | 2% BSA |
| Rabbit Lactoferrin (ab135710; Abcam) | 1/500 | 2% BSA |
| Rabbit Nuclear factor erythroid 2-related factor 2 (ab62352; Abcam) | 1/500 | 2% milk |
| Rabbit Acyl-CoA Synthetase Long Chain Family Member 4 (ab155282; Abcam) | 1/10000 | 2% milk |
| Goat light-chain subunit of Cystine/glutamate transporter (SAB2500951; Merck) | 1/500 | 2% milk |
| Rabbit Glutathione peroxidase 4 (SAB4300725; Merck) | 1/500 | 2% milk |
| Rabbit 4-Hydroxynonenal (ab46545; Abcam) | 1/1000 | 2% milk |
| Mouse HRP-conjugated β-actin (clone AC-15, ab49900; Abcam) | 1/25000 | 2% milk |

**Table S2:** Expression levels of proteins involved in iron metabolism and ferroptosis in the medial temporal lobe of cognitively normal (CN) and Alzheimer’s disease (AD) subjects. Data are normalised to actin and presented as mean ± standard deviation. Significance was set at a threshold of p ≤ 0.05, with * and *** being p < 0.05 and p < 0.005, respectively. Proteins that are expressed significant differently between CN and AD are in bold. Abbreviations: Ferritin light-chain (FTL), Ferritin heavy-chain (FTH), Transferrin receptor (TfR), Divalent metal transporter 1 (DMT1), Iron Responsive Element Binding Protein 2 (IREB2), Ceruloplasmin (Cp), Ferroportin (Fpn), Heme-oxygenase 1 (HO-1), Melanotransferrin (MTf), Lactotransferrin (LTf), Nuclear factor erythroid 2-related factor 2 (Nrf2), Acyl-CoA Synthetase Long Chain Family Member 4 (ACSL4), light-chain subunit of cystine/glutamate transporter (xCT), Glutathione peroxidase 4 (GPX4) and 4-Hydroxynonenal (4-HNE).

|  | CN (n) | AD (n) | p-value | t-value (df) |
| --- | --- | --- | --- | --- |
| FTL | 0.371 ± 0.173 (5) | 0.736 ± 0.317 (6) | **0.0420*** | 2.421 (7.943) |
| FTH | 1.149 ± 0.143 (5) | 2.040 ± 0.692 (7) | **0.0137*** | 3.311 (6.705) |
| TfR | 0.185 ± 0.062 (6) | 0.159 ± 0.059 (7) | 0.4524 | 0.780 (10.55) |
| DMT1 | 3.407 ± 0.559 (7) | 2.517 ± 0.868 (6) | 0.0617 | 2.158 (8.316) |
| IREB2 | 0.956 ± 0.381 (7) | 1.128 ± 0.504 (7) | 0.4848 | 0.723 (11.17) |
| Cp | 0.220 ± 0.069 (7) | 0.461 ± 0.198 (7) | **0.0173*** | 3.052 (7.418) |
| Fpn | 0.740 ± 0.246 (6) | 0.502 ± 0.093 (6) | 0.0655 | 2.219 (6.401) |
| HO-1 | 0.312 ± 0.118 (7) | 0.294 ± 0.079 (7) | 0.7399 | 0.341 (10.44) |
| MTf | 1.787 ± 0.295 (7) | 1.687 ± 0.364 (7) | 0.5815 | 0.567 (11.50) |
| LTf | 0.498 ± 0.196 (5) | 0.592 ± 0.159 (7) | 0.4029 | 0.886 (7.551) |
| Nrf2 | 0.263 ± 0.084 (6) | 0.206 ± 0.052 (7) | 0.1908 | 1.427 (8.123) |
| ACSL4 | 0.701 ± 0.284 (7) | 0.688 ± 0.167 (7) | 0.9217 | 0.101 (9.682) |
| xCT | 0.420 ± 0.093 (5) | 0.538 ± 0.065 (6) | **0.0486*** | 2.385 (6.977) |
| GPX4 | 3.254 ± 0.889 (7) | 3.083 ± 0.867 (7) | 0.7221 | 0.364 (11.99) |
| 4-HNE | 2.421 ± 0.264 (6) | 3.183 ± 0.141 (6) | **0.0003***** | 6.237 (7.625) |

**Table S3**: Metabolite concentrations quantified from ^1^H-NMR spectra of aqueous and organic extracts of medial temporal cortex tissue of cognitively normal (CN) and Alzheimer’s disease (AD) subjects. ~Resonances are observed from a variety of lipids in each lipid class which leads to several overlapping resonances and only resonances used for quantification are detailed. ^#^Cholesterol also has many resonances and again only the resonance used for quantification is noted. Data are normalised to protein levels and presented as mean ± standard deviation. Significance was set at p ≤ 0.05, with *, ** and *** being p < 0.05, 0.01 and p < 0.005, respectively. Metabolites levels that are significantly different between CN and AD are in bold. Abbreviations: singlet (s), doublet (d), triplet (t), quartet (q), multiplet (m), doublet of doublets (dd) and polyunsaturated fatty acids (PUFA).

| Metabolite | Chemical shift (δ/ppm), resonance multiplicities | Assignment | Control (n)  (μmol/g) | Alzheimer’s disease (n)  (μmol/g) | p-value (t,df) |
| --- | --- | --- | --- | --- | --- |
| Acetate | 1.92, s | CH_3_ | 1.22 ± 0.44 (7) | 1.21 ± 0.37 (6) | 0.998 (0.02,11) |
| Alanine | 1.48, d; 3.76, q | CH_3_; CH | 2.41 ± 1.02 (7) | 2.03 ± 0.74 (6) | 0.453 (0.78,10.8) |
| Aspartate | 2.68, dd; 2.78, dd; 3.90, dd | 3-CH; 3-CH’; 2-CH | 2.13 ± 1.04 (7) | 2.38 ± 0.79 (6) | 0.634 (0.49,10.9) |
| Carnitine | 2.45, dd; 3.24, s; 3.41, m; 4.56, m | 2-CH_2_; (CH_3_)_3_; 4-CH_2_; 3-CH | 0.70 ± 0.32 (7) | 0.55 ± 0.20 (6) | 0.181 (1.43,10.9) |
| Choline | 3.18, s; 3.51, m; 4.07, m | CH_3_; 1-CH_2_; 2-CH_2_ | 0.65 ± 0.40 (7) | 0.49 ± 0.18 (6) | 0.361 (0.97,8.5) |
| Creatine | 3.04, s; 3.92, s | CH_3_; CH_2_ | 8.29 ± 2.73 (7) | 5.64 ± 1.44 (6) | 0.052 (2.23,9.3) |
| Formate | 8.45, s | CH | 6.73 ± 4.46 (7) | 3.91 ± 2.29 (6) | 0.177 (1.46,9.2) |
| GABA | 1.90, m; 2.30, t; 3.01, t | 3-CH2, 2-CH2, 4-CH | 1.60 ± 0.75 (7) | 0.86 ± 0.34 (6) | 0.052 (2.23,8.7) |
| Glutamate | 2.07, m; 2.13, m; 2.34, m; 3.75, dd | 3-C**H**H’; 3-CH**H’;** 4-CH; 2-CH | 9.17 ± 3.73 (7) | 8.01 ± 2.07 (6) | 0.499 (0.70,9.6) |
| Glutamine | 2.13, m; 2.45, m; 3.76, t | 3-CH_2_; 4-CH_2_; 2-CH | 6.87 ± 2.66 (7) | 3.26 ± 1.23 (6) | **0.011** (3.21,8.7) |
| Glycerol | 3.56, dd; 3.65, dd; 3.78, m | 1/3-C**H**H’; 1/3-CH**H**’; 2-CH | 5.38 ± 2.17 (7) | 3.37 ± 0.82 (6) | 0.054 (2.26,7.9) |
| Glycine | 3.55, s | CH_2_ | 2.25 ± 0.84 (7) | 1.93 ± 0.66 (6) | 0.461 (0.76,10.9) |
| Hypoxanthine | 8.19, s; 8.21, s | 2-CH; 8-CH | 1.16 ± 0.37 (7) | 0.78 ± 0.18 (6) | **0.0397** (2.41,8.9) |
| Inosine | 3.83, dd; 3.90, dd; 4.26, dd; 4.44, t; 4.76, t; 6.10, d; 8.22, s; 8.35, s | 5’-C**H**H’; 5’-CH**H’**; 4’-CH; 3’-CH, 2’-CH; 1’-CH; 2-CH; 8-CH | 0.43 ± 0.24 (7) | 0.19 ± 0.16 (6) | 0.053 (2.18,10.4) |
| Isoleucine | 0.92, t; 0.98, d; 1.16, m; 1.45, m; 1.97, m; 3.66, d | 5-CH_3_; 6-CH_3_; 4-C**H**H’; 4-CH**H**’; 3-CH; 2-CH | 0.36 ± 0.22 (7) | 0.43 ± 0.24 (6) | 0.619 (0.51,10.3) |
| Lactate | 1.33, d; 4.1, q | CH_3_; CH | 36.86 ±14.98 (7) | 36.86 ± 14.98 (6) | 0.220 (1.31,10.2) |
| Leucine | 0.96, d; 0.97 d; 1.66, m; 1.70, m; 1.73, m; 3.73, t | CH_3_; CH_3_; 3-C**H**H’; 4-CH; 3-CH**H**’; 2-CH | 0.82 ± 0.42 (7) | 27.91 ± 9.41 (6) | 0.428 (0.83,9.2) |
| Myo-inositol | 3.27, t; 3.53, dd; 3.62, t; 4.06, t | 5-CH; 1-CH and 3-CH; 4-CH and 6-CH; 2-CH | 7.32 ± 3.13 (7) | 6.11 ± 0.86 (6) | 0.360 (0.98,7.0) |
| N-Acetyl-aspartate | 2.01, s; 2.49, dd; 2.69, dd; 4.39, dd | CH_3_; 3-C**H**H’; 3-CH**H**’; 2-CH | 6.18 ± 2.89 (7) | 2.70 ± 1.47 (6) | **0.021** (2.80,9.2) |
| Phenylalanine | 3.19, m; 3.98, m; 4.00, dd; 7.32, d;  7.36, m; 7.42, m | 2-C**H**H’; 2-CH**H**’; 3-CH, *ortho*-CH; *para*-CH; *meta*-CH | 0.44 ± 0.20 (7) | 0.52 ± 0.23 (6) | 0.518 (0.67,10.0) |
| Succinate | 2.40, s | CH_2_ | 0.48 ± 0.25 (7) | 0.28 ± 0.09 (6) | 0.090 (1.93,7.8) |
| Tyrosine | 3.02, dd; 3.17, dd; 3.92, dd;  6.88, d; 7.17, d | 2-C**H**H’; 2-CH**H**’; 3-CH; *ortho*-CH; *para*-CH | 0.26 ± 0.20 (7) | 0.33 ± 0.16 (6) | 0.518 (0.67,10.9) |
| Valine | 0.99, d; 1.04, d; 2.25, m; 3.60, d | CH_3_; CH_3_;  3-CH; 2-CH | 0.55 ± 0.27 (7) | 0.69 ± 0.36 (6) | 0.482 (0.73,9.2) |
| Fatty acids~ | 0.88 – 0.98, m (overlapping t and cholesterol contributions were subtracted) | CH_3_ | 100.80 ± 42.37 (7) | 58.68 ± 33.33 (6) | 0.071 (2.00,11) |
| PUFAs~ | 2.87 – 2.93, m | -CH=CH-C**H_2_**-CH=CH | 14.53 ± 6.80 (7) | 8.91 ± 4.35 (7) | 0.095 (1.84,10.2) |
| Triglycerides~ | 5.12, m | -COCH_2_-C**H**-CH_2_CO- | 80.45 ± 35.20 (7) | 51.48 ± 33.40 (6) | 0.157 (1.52,10.9) |
| Cholesterol^#^ | 0.84, s | 18-C**H_3_** | 17.66 ± 7.01 (7) | 14.04 ± 10.09 (7) | 0.452 (0.78,10.7) |

**Figure S1** – Representative western blots of proteins that were at comparable expression levels in cognitively normal (CN) and Alzheimer’s disease (AD). Abbreviations are: transferrin-receptor (TfR), Iron Responsive Element Binding Protein 2 (IREB2), heme-oxygenase-1 (HO-1), melanotransferrin (MTf), lactoferrin (LTf), Nuclear factor erythroid 2-related factor 2 (Nrf2), Acyl-CoA Synthetase Long Chain Family Member 4 (ACSL4) and glutathione peroxidase 4 (GPX4).


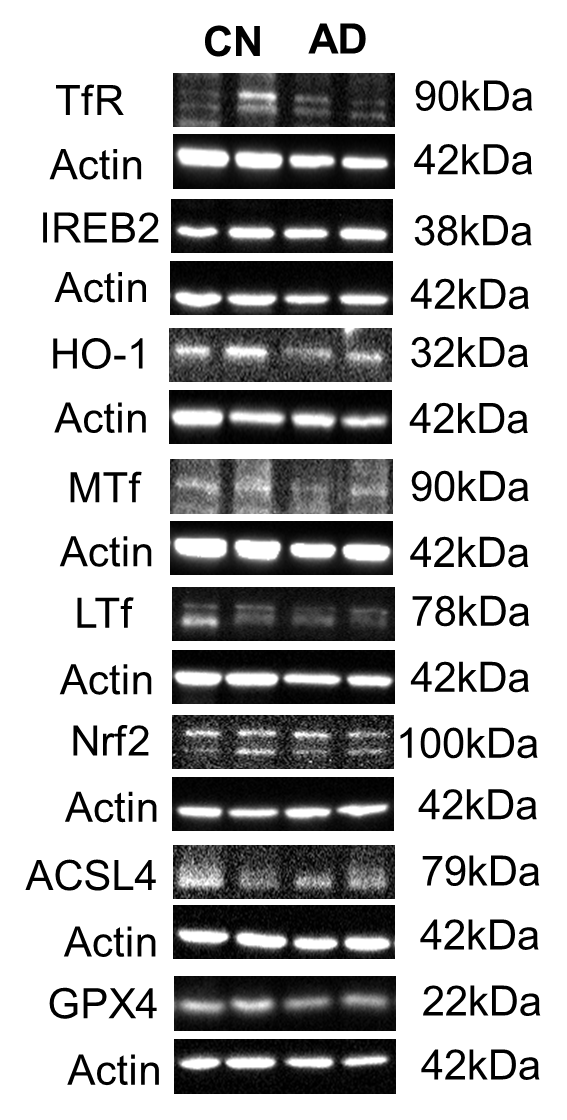

Supplement: Multimedia component 1 [file mmc1.docx]
